# A Novel SXT/R391 Integrative and Conjugative Element Carries Two Copies of the *bla*_NDM-1_ Gene in Proteus mirabilis

**DOI:** 10.1128/mSphere.00588-21

**Published:** 2021-08-11

**Authors:** Jintao He, Long Sun, Linghong Zhang, Sebastian Leptihn, Yunsong Yu, Xiaoting Hua

**Affiliations:** a Department of Infectious Diseases, Sir Run Run Shaw Hospital, Zhejiang University School of Medicine, Hangzhou, China; b Key Laboratory of Microbial Technology and Bioinformatics of Zhejiang Province, Hangzhou, China; c Regional Medical Center for National Institute of Respiratory Diseases, Sir Run Run Shaw Hospital, School of Medicine, Zhejiang University, Hangzhou, China; d Department of Clinical Laboratory, Hangzhou Women’s Hospital, Hangzhou Maternity and Child Health Care Hospital, Hangzhou, China; e Zhejiang University grid.13402.34Zhejiang University-University of Edinburgh Institute, Haining, China; Antimicrobial Development Specialists, LLC

**Keywords:** *Proteus mirabilis*, SXT/R391, ICE, tandem copies, *bla*_NDM-1_, IS*CR1*

## Abstract

The rapid spread of the *bla*_NDM-1_ gene is a major public health concern. Here, we describe the multidrug-resistant Proteus mirabilis strain XH1653, which contains a novel SXT/R391 integrative and conjugative element (ICE), harboring two tandem copies of *bla*_NDM-1_ and 21 other resistance genes. XH1653 was resistant to all antibiotics tested, apart from aztreonam. Whole-genome data revealed that two copies of *bla*_NDM-1_ embedded in the IS*CR1* element are located in HS4 of the novel ICE, which we named ICE*Pmi*ChnXH1653. A circular intermediate of ICE*Pmi*ChnXH1653 was detected by PCR, and conjugation experiments showed that the ICE can be transferred to the Escherichia coli strain EC600 with frequencies of 1.5 × 10^−7^. In the recipient strain, the ICE exhibited a higher excision frequency and extrachromosomal copy number than the ICE in the donor strain. We also observed that the presence of ICE*Pmi*ChnXH1653 has a negative impact on bacterial fitness and leads to changes in the transcriptome of the host. *In vitro* evolution experiments under nonselective conditions showed that the two tandem copies of the IS*CR1* element and the IS*Vsa3* element can be lost during repeated laboratory passage. This is the first report of a novel SXT/R391 ICE carrying two tandem copies of *bla*_NDM-1_, which also illustrates the role that ICEs may play as platforms for the accumulation and transmission of antibiotic resistance genes.

**IMPORTANCE** The occurrence of carbapenemase-producing Proteus mirabilis, especially those strains producing NDM-1 and its variants, is a major public health concern worldwide. The integrative conjugative element (ICE) plays an important role in horizontal acquisition of resistance genes. In this study, we characterized a novel SXT/R391 ICE from a clinical P. mirabilis isolate that we named ICE*Pmi*ChnXH1653, which contains two tandem copies of the carbapenemase gene *bla*_NDM-1_. We performed an integrative approach to gain insights into different aspects of ICE*Pmi*ChnXH1653 evolution and biology and observed that ICE*Pmi*ChnXH1653 obtained the carbapenemase gene *bla*_NDM-1_ by IS*CR1*-mediated homologous recombination. Our study reveals that the transmission of *bla*_NDM-1_ by IS*CR1* elements or ICEs may be an important contributor to the carbapenem resistance development across species, which could improve our understanding of horizontal gene transfer in clinical environments.

## INTRODUCTION

The Gram-negative bacillus Proteus mirabilis is emerging as an increasingly important pathogen in nosocomial infections, particularly in urinary tract infections (1). Due to intrinsic resistance to nitrofurantoin, polymyxin, and tigecycline, the occurrence of carbapenemase-producing P. mirabilis is of particular concern; in particular, strains producing the New Delhi metallo-β-lactamase 1 (NDM-1) make treatment extremely difficult (2, 3). NDM-1, an Ambler class B β-lactamase, is the main type of carbapenemase, conferring resistance to almost all β-lactams except monobactams (4). Global dissemination of NDM-1 is a major public health problem (5, 6). *bla*_NDM-1_ is a chimeric gene that has arisen by a gene fusion event linking the aminoglycoside resistance gene *aphA6* to a preexisting metallo-β-lactamase (MBL) gene and is frequently located downstream of a complete or truncated copy of IS*Aba125* (7). The *bla*_NDM-1_ gene has been found in various genetic contexts including prophages (8) but is most often associated with other mobile genetic elements that are responsible for its evolution and rapid transmission (9). For instance, IS*CR*s (insertion sequences with a common region) are often found in the vicinity of *bla*_NDM-1_ and considered one of the routes of the fusion of *bla*_NDM-1_ and also involved in the mobilization of *bla*_NDM-1_ (7, 9–11). In addition, horizontal dissemination of *bla*_NDM-1_ among diverse species can occur via mobile DNA vectors, mostly conjugative plasmids and integrative conjugative elements (ICEs) (12, 13).

ICEs are self-transmissible mobile genetic elements which encode modules that facilitate integration/excision, conjugative transfer, and maintenance (14). The element can therefore be excised from the host chromosome to form a circular intermediate that can be transferred to a recipient cell via conjugation (15). The SXT/R391 family, one of the largest families of ICEs, has a highly conserved core of sequences that mediate the site-specific integration into the 5′ end of the *prfC* gene (14, 16). Except for a conserved backbone, SXT/R391 ICEs frequently contain hot spots (HS1 to HS5) and variable regions (VRI to VRV) that carry genes for antimicrobial resistance and metal tolerance (17, 18). Many SXT/R391 ICEs have been found in P. mirabilis, contributing to the dissemination of antimicrobial resistance genes (ARGs) including the cephalosporinase gene *bla*_CMY-2_ (19–21). Most recently, an SXT/R391 ICE carrying the carbapenemase gene *bla*_NDM-1_ embedded within a truncated Tn*125* was identified in Proteus vulgaris (13).

In this study, we characterized a novel SXT/R391 ICE from a P. mirabilis isolate, named ICE*Pmi*ChnXH1653, which contains two tandem copies of *bla*_NDM-1_ and 21 other ARGs. An integrative approach, combining bacterial conjugation tests, fitness assays, experimental evolution, genomics, and transcriptomics, allowed us to gain insights into diverse aspects of evolution and biology of ICE*Pmi*ChnXH1653 (22).

## RESULTS

### Characterization of *bla*_NDM-1_-bearing P. mirabilis strain XH1653.

**FIG 1:**
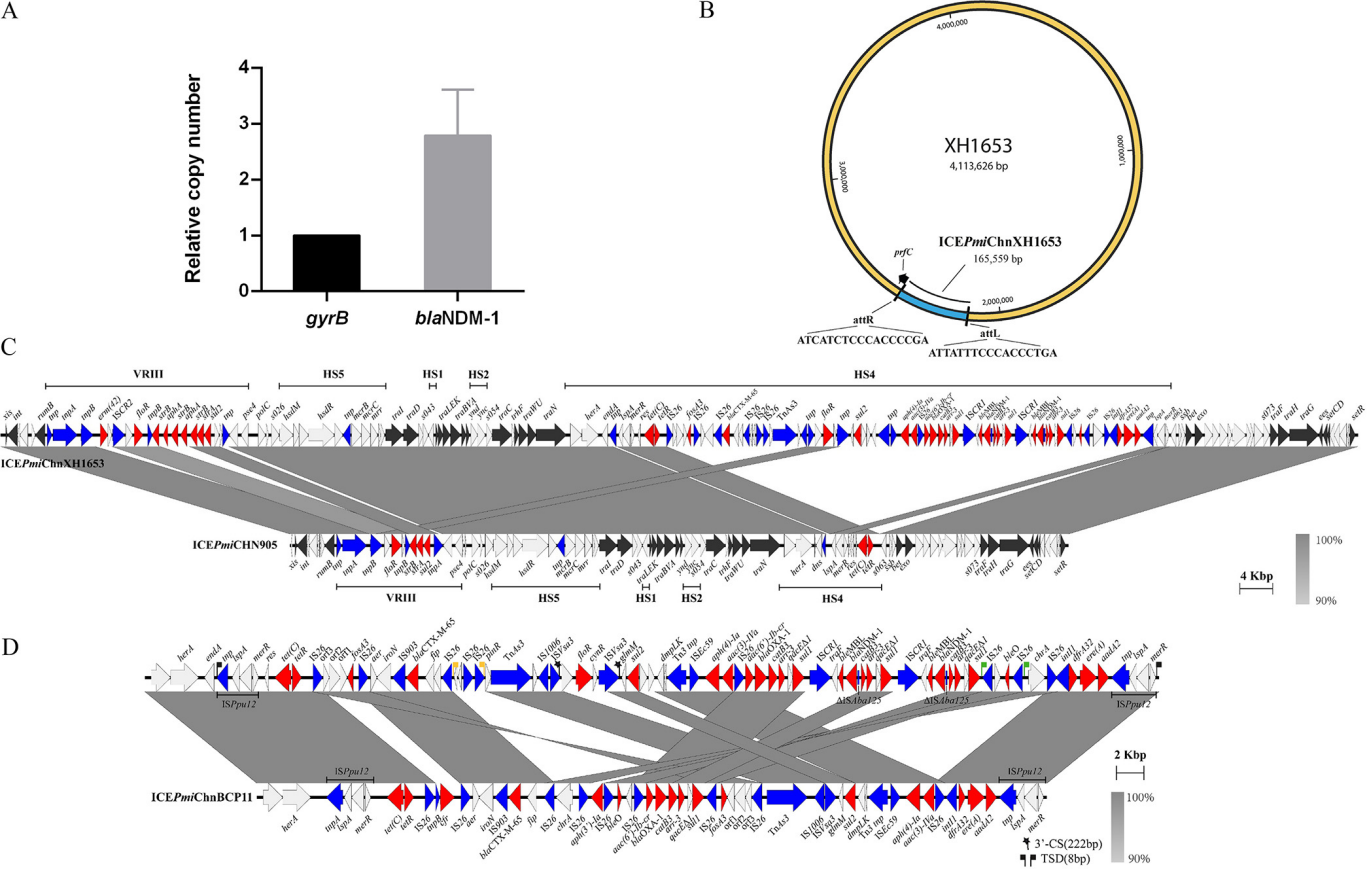
Characterization of *bla*_NDM-1_-bearing P. mirabilis strain XH1653. (A) Relative quantification of *bla*_NDM-1_. *gyrB* was used as control. (B) Graphical map of the XH1653 chromosome. (C and D) Genetic structure of ICE*Pmi*ChnXH1653. (C) ICE*Pmi*ChnXH1653 compared with ICE*Pmi*CHN905 (KX243412.1). (D) HS4 of ICE*Pmi*ChnXH1653 compared with HS4 of ICE*Pmi*ChnBCP11 (MG773277). ARGs are in red, transposase or integrase genes are in blue, core genes are in black, and other genes are in white. Different colors of target site duplication (TSD) in panel D represent different TSD sequences.

While being intrinsically resistant to tetracycline and colistin, the P. mirabilis strain XH1653 exhibited resistance to all tested antimicrobials with the exception of aztreonam, thus defining the strain as multi-/extensively drug resistant (MDR/XDR). The strain contains a single circular chromosome with a size of 4,113,626 bp (GC content 39.2%). We identified multiple ARGs including those for β-lactams (*bla*_NDM-1_, *bla*_CTX-M-65_, *bla*_OXA-1_), fluoroquinolone [*aac(6′)-Ib-cr*], fosfomycin (*fosA3*), tetracycline [*tet(C)*, *tet(J)*], aminoglycosides [*aadA2*, *aph(4)-Ia*, *strB*, *strA*, *aac(6′)-Ib-cr*, *aphA*, *aac(3)-IVa*], sulfamethoxazole (*sul1*, *sul2*), trimethoprim (*dfrA32*), phenicol (*catB3*, *floR*, *catA4*), rifamycin (*arr-3*), macrolide [*ere(A)*, *erm(42)*], and bleomycin (*ble*_MBL_, *ble*O). Interestingly, we found that XH1653 contains two copies of the carbapenemase gene *bla*_NDM-1_. To corroborate this finding, we employed real-time quantitative PCR (qPCR) to determine the number of copies of *bla*_NDM-1_ per cell, which confirmed the presence of multiple copies of *bla*_NDM-1_ (2.79 ± 0.67 copies/cell) (Fig. 1A). Further sequence analysis showed that all ARGs, with the exception of *catA4* and *tet(J)*, were located on a novel integrative conjugative element, designated ICE*Pmi*ChnXH1653 according to the proposed nomenclature of ICEs (14). The name of this new ICE had been registered with Adam Roberts in Liverpool, United Kingdom, as Tn*7349* (23).

### Genetic structure of ICE*Pmi*ChnXH1653.

ICE*Pmi*ChnXH1653 has a length of 165,609 bp (bases 2138935 to 2304493 in XH1653) and a GC content of 50.0%. It was found integrated into the 5′ end of the *prfC* gene, a common insertion site for ICEs in the SXT/R391 family (Fig. 1B). ICE*Pmi*ChnXH1653 consisted of a highly conserved backbone involved in essential functions of SXT/R391 ICEs, such as integration (*int* and *xis*), mating pair formation (*traLEKBVA*), exclusion determination (*traG* and *ees*), DNA recombination (*bet* and *exo*), and regulation (*setR*) (24). BLAST analysis showed that ICE*Pmi*ChnXH1653 had 100% nucleotide identity at 98% coverage to ICE*Pmi*CHN905 that was found in the P. mirabilis strain MD20140905 isolated from stool samples from diarrhea patients in Beijing, China, in 2014, with two region divergences in VRIII and HS4 (Fig. 1C) (25), indicating that ICE*Pmi*ChnXH1653 might have a common origin with ICE*Pmi*CHN905.

### Characterization of VRIII and HS4 region of ICE*Pmi*ChnXH1653.

ICE*Pmi*ChnXH1653 harbored two multidrug resistance (MDR) regions in VRIII and HS4, respectively. VRIII contained six ARGs, including *erm(42)*, *floR*, *strB* (three copies), *aphA* (two copies), *strA*, and *sul2*. Structural comparison showed that the formation of this MDR cluster in VRIII was likely due to the abundance of transposases and IS*CR2* elements.

The MDR region HS4 is 73.75 kb and harbors 19 ARGs coding for β-lactam, fluoroquinolone, fosfomycin, tetracycline, aminoglycoside, sulfamethoxazole, trimethoprim, streptomycin, phenicol, rifamycin, macrolide, and bleomycin resistance, which are clustered together in an IS*Ppu12*-mediated composite transposon flanked by the 8-bp target site duplication (TSD) TAAAGAAA. According to a BLAST analysis, HS4 in ICE*Pmi*ChnXH1653 had 99.92% nucleotide identity at 82% coverage to the HS4 in ICE*Pmi*BCP11 that was found in the P. mirabilis strain BCP11 isolated from a fecal swab of a diseased pig with diarrhea in Sichuan Province of China in November 2016, and with the exception of *cfr*, it carried all the ARGs that were found in ICE*Pmi*BCP11. The HS4 region of ICE*Pmi*ChnXH1653 also has a similar genetic environment as HS4 of ICE*Pmi*BCP11, which suggests that these two ICEs isolated from China share an IS*Ppu12*-mediated region (Fig. 1D). However, there are major differences between them: two copies of the carbapenemase gene *bla*_NDM-1_ and the chloramphenicol ARG *floR* were detected in the HS4 region of ICE*Pmi*ChnXH1653. Further analysis of the insertion sequences surrounding *bla*_NDM-1_ revealed two tandem copies of an IS*CR1*element (IS*CR1*-*traF*-*ble*_MBL_-*bla*_NDM-1_-ΔIS*Aba125*-*catB3*-*arr-3*-*qacE*Δ*1*-*sul1*). The sequence of the IS*CR1* element in the HS4 region of ICE*Pmi*ChnXH1653 showed 99.95% nucleotide identity to plasmid pNDM-PM58 from P. mirabilis (GenBank accession no. KP662515.1). Moreover, the two tandem copies of the IS*CR1* element were also seen in Escherichia coli Y5 (99.98% nucleotide identity; GenBank accession no. CP013483) that was reported by our lab in 2016 (26). The *floR* gene was flanked by IS*Vsa3* elements with a 222-bp 3′-conserved segment (3′-CS), indicating that IS*Vsa3* promotes the dissemination of *floR.* Two copies of IS*26* adjacent to *ble*O are in the same orientation, while another IS*26* and two genes (encoded recombinase family protein and transposase, respectively) lie in the opposite orientation, flanked by identical 8-bp TSDs (GTTCATAC; CGCCGGTG). This indicates that IS*26* is involved in the accumulation of resistance genes and the rearrangement of multidrug resistance regions.

### Transfer ability of ICE*Pmi*ChnXH1653.

**FIG 2:**
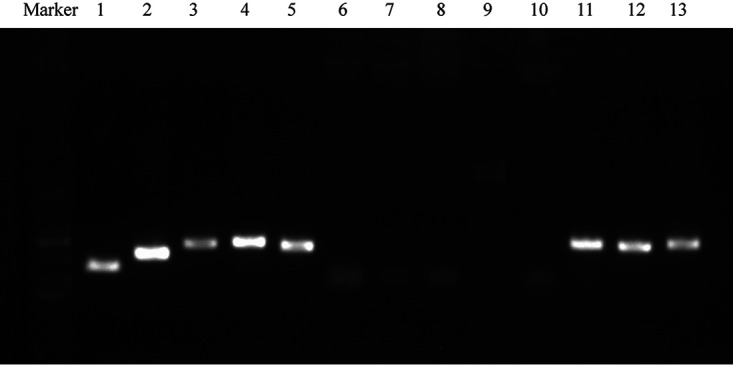
PCR electrophoresis map of XH1814 (lanes 1 to 5), EC600 (lanes 6 to 10), and XH1653 (lines 11 to 13). Lanes 1 and 6 were the *attL* fragment; lanes 2 and 7 were the *attR* fragment; lanes 3, 8, and 11 were the *int* fragment; lanes 4, 9, and 12 were the *bla*_NDM-1_ fragment; lanes 5, 10, and 13 were the fragment of the circular ICE.

To test this ability to transfer ICE*Pmi*ChnXH1653, conjugation experiments were performed, with the P. mirabilis strain XH1653 as donor and the rifampin-resistant strain E. coli EC600 as recipient. ICE*Pmi*ChnXH1653 was successfully transferred to E. coli EC600 with a frequency of 1.5 × 10^−7^ transconjugants per recipient cell and chromosomally integrated into the 5′ end of *prfC*. The positive transconjugant, subsequently referred to as XH1814, was confirmed by matrix-assisted laser desorption ionization–time of flight mass spectrometry (MALDI-TOF MS) and PCR detection of *int*, *attL*, *attR*, and the carbapenemase gene *bla*_NDM-1_. Antimicrobial susceptibility testing showed XH1814 acquired resistance to all antimicrobials tested, apart from ciprofloxacin (Table 1). Excision of the ICE*Pmi*ChnXH1653 and the presence of a circular form were analyzed using PCR with primers LE4 and RE4 (24). The analysis confirmed occurrence of the circular ICE in XH1653 and XH1814 (Fig. 2), indicating that ICE*Pmi*ChnXH1653 could form circular intermediates.

**TABLE 1:** Antimicrobial susceptibilities of P. mirabilis XH1653, E. coli EC600, and the transconjugants E. coli XH1814 and E. coli XH1815

Strain	MIC (mg/liter) of drug^*a*^:						
	MEM	IPM	GEN	FEP	CIP	TET	SXT
XH1653	16	32	32	32	64	64	>32
EC600	0.03	0.25	1	0.125	0.25	0.5	≤0.25
XH1814	8	4	32	32	0.25	32	>32
XH1815	0.015	0.25	32	8	0.5	32	>32

a Abbreviations: MEM, meropenem; IPM, imipenem; GEN, gentamicin; FEP, cefepime; CIP, ciprofloxacin; TET, tetracycline; SXT, trimethoprim-sulfamethoxazole.

**FIG 3:**
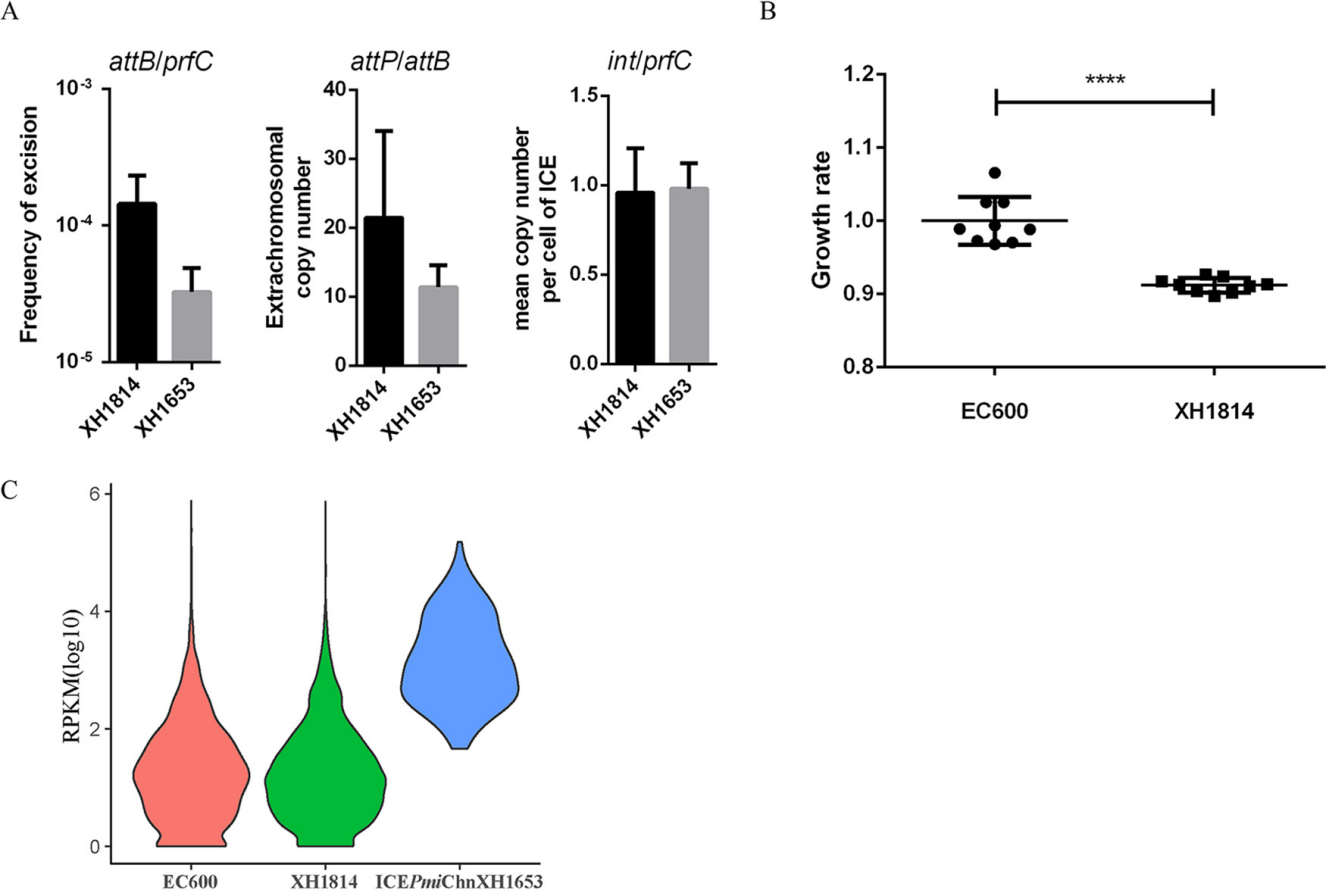
Characterization of the biology of ICE*Pmi*ChnXH1653. (A) Frequency of excision, extrachromosomal copy number of the ICE, and the mean copy number per cell of ICE*Pmi*ChnXH1653 in XH1814 and XH1653. (B) Growth rates of XH1814 and EC600. The experiment was repeated in triplicate. Representative results of three independent experiments are shown, and the data are the mean ± standard deviation (SD). ****, *P* < 0.0001 (Student’s *t* test). (C) The gene expression value (RPKM) in EC600, XH1814 (excluding the genes in ICE*Pmi*ChnXH1653), and ICE*Pmi*ChnXH1653 in XH1814.

To investigate the biology of ICE*Pmi*ChnXH1653, we evaluated three dynamic factors in both the ancestor strain and the recipient strain. Real-time quantitative PCR assays were developed to determine the percentages of *int*/*prfC* (mean copy number per cell), *attB*/*attP* (extrachromosomal copy number of the ICE), and *attB/prfC* (the frequency of excision) in a culture (Fig. 3A). The ratio of *int*/*prfC* was found to be 0.96 ± 0.20 for XH1814 and 0.98 ± 0.11 for XH1653, as expected for a single copy of an ICE integrated in the chromosome *prfC* target. The results of *attB*/*attP* showed that ICE*Pmi*ChnXH1653 in XH1814 exhibited a relatively higher excision frequency than the ICE in XH1653; this might indicate that the ICE is not as stable in E. coli as it is in the original host. We also found that both XH1814 and XH1653 had multiple copies of extrachromosomal ICEs (21 ± 10 and 11 ± 3, respectively), suggesting that the circular intermediate of ICE*Pmi*ChnXH1653 is capable of replicating in a small subset of the cell population.

### Presence of ICE*Pmi*ChnXH1653 influences host fitness and transcriptome.

To estimate the fitness cost of ICE carriage, we compared growth rates of the transconjugant XH1814 and the ICE-free recipient strain EC600 in Mueller-Hinton (MH) liquid medium. XH1814 containing ICE*Pmi*ChnXH1653 exhibited a significantly decreased growth rate compared to the strain without the ICE, EC600 (Fig. 3B), indicating that ICE*Pmi*ChnXH1653 confers a fitness cost on the host.

To better understand the molecular basis for the decrease in fitness due to ICE*Pmi*ChnXH1653 in EC600, we performed transcriptomic analyses of the strain with and without the ICE (XH1814 versus EC600) using RNA sequencing (RNA-Seq). In comparison to strain EC600, a total of 22 genes were differentially expressed in XH1814 (false-discovery rate [FDR] < 0.05). Among them, nine genes were upregulated, and 13 genes were downregulated (see Table S1 in the supplemental material). The upregulated genes were involved in lipid metabolism and amino acid metabolism, and the downregulated genes were involved in the metabolic pathway, replication and repair pathway, and quorum sensing pathway. Increased transcription of genes encoding 4-oxalomesaconate tautomerase (DK885_16125), bifunctional aldehyde dehydrogenase (DK885_12690), hydrogenase-4 component J (DK885_06720), and galactose-proton symporter (DK885_04375) was noticeable, whereas genes for multidrug resistance protein (MdtL), guanine/hypoxanthine permease (GhxQ), and cold shock protein (CspB) were expressed at lower levels in XH1814. We also compared gene expression levels of EC600 and XH1814 using the average reads per kilobase per million mapped reads (RPKM), with no significant difference between the detectable levels. However, the average RPKM of ICE in XH1814 was higher than the average RPKM of EC600 or XH1814 (*P < *0.05) (Fig. 3C).

10.1128/mSphere.00588-21.1 TABLE S1 Genes changed significantly in transcriptome. Download Table S1, PDF file, 0.1 MB. Copyright © 2021 He et al. 2021 He et al. https://creativecommons.org/licenses/by/4.0/ This content is distributed under the terms of the Creative Commons Attribution 4.0 International license.

### XH1814 lost carbapenem resistance following repeated laboratory passage.

**FIG 4:**
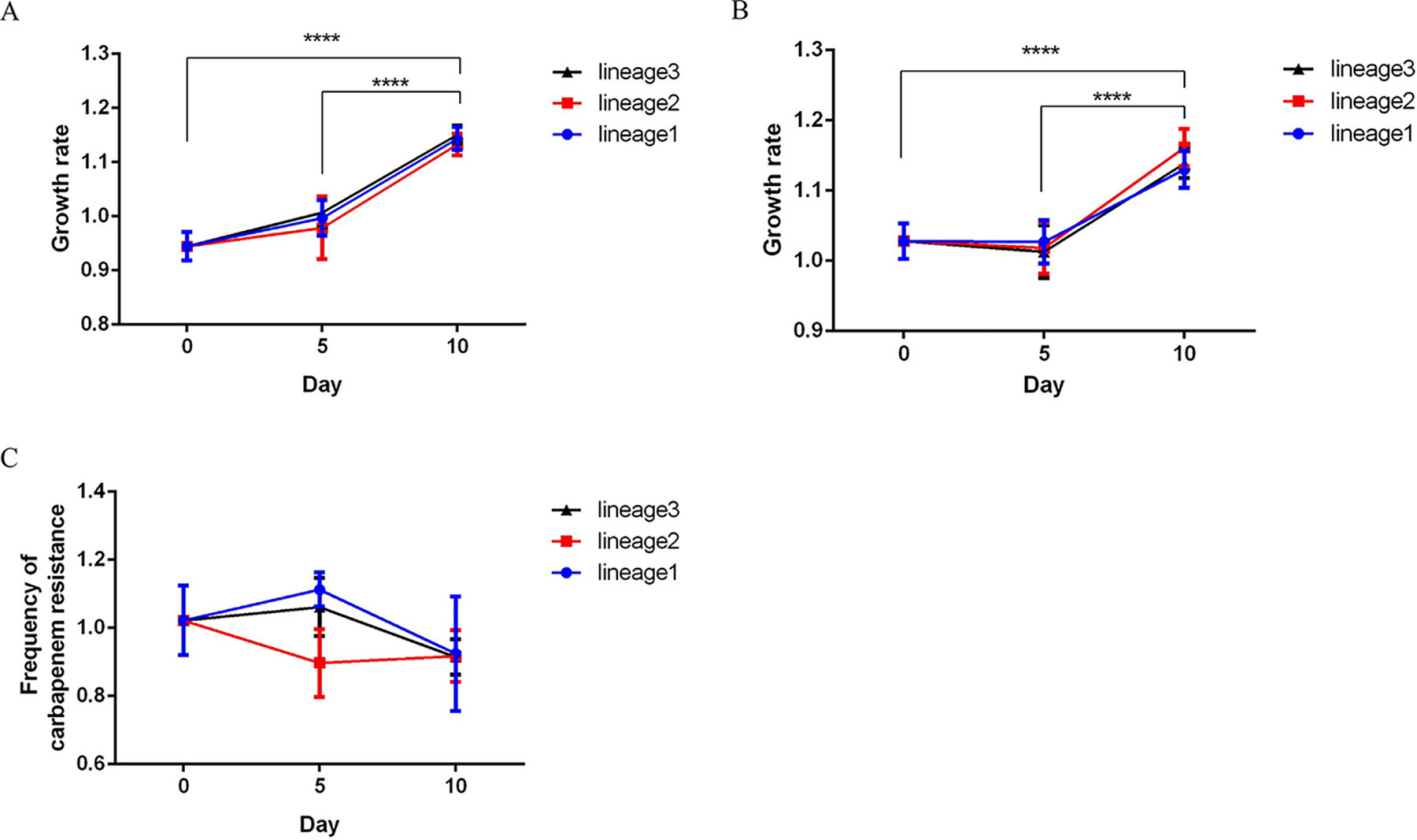
Characterization of the evolution of ICE*Pmi*ChnXH1653. (A and B) Line charts of experimental evolution of XH1814 (A) and EC600 (B) in MH broth without antibiotics. (C) Line chart of the frequency of XH1814 harboring the carbapenem resistance. The data represent the averages from three experiments. Standard errors of the means are indicated. The *P* values represent the averages from three lineages and were determined by two-tailed Student’s *t* tests. ****, *P* < 0.0001.

**FIG 5:**
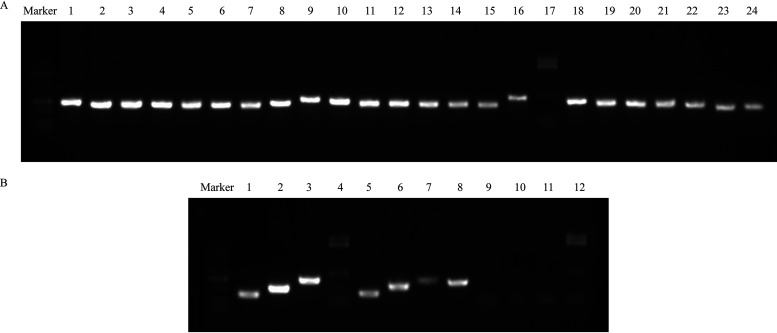
PCR electrophoresis map. (A) Detecting the *bla*_NDM-1_ fragment of XH1814D10. (B) Detecting the *attL*, *attR*, *int*, and *bla*_NDM-1_ fragments of XH1815 (lanes 1 to 4), XH1814 (lanes 5 to 8), and EC600 (lanes 9 to 12). Lanes 1, 5, and 9 were the *attL* fragment; lanes 2, 6, and 10 were the *attR* fragment; lanes 3, 7, and 11 were the *int* fragment; lanes 4, 8, and 12 were the *bla*_NDM-1_ fragment.

Given the apparent cost of the ICE*Pmi*ChnXH1653 carriage, we performed experimental evolution experiments with the transconjugant XH1814 and the ICE-free recipient strain EC600 as a control to identify the putative emergence of compensatory mechanisms associated with ICE carriage. We observed that the strain on day 10, “XH1814D10,” had a significantly faster growth than XH1814 and the strain on day 5 (Fig. 4A). However, the ICE-free control strain also showed evolutionary adaptations with significantly faster growth than the strain on day 0 and day 5 (Fig. 4B). As compensatory mutations may not be associated with ICE carriage, we chose not to trace compensatory mutations on the whole-genome level. When testing the presence of ICE*Pmi*ChnXH1653 in “XH1814D10” using PCR with *bla*_NDM-1_ primers, we observed that the *bla*_NDM-1_ gene was lost while still retaining the ICE (Fig. 5A and B). This strain, named XH1815, had become susceptible to imipenem and meropenem (Table 1). Also, the loss of carbapenem resistance was observed, albeit at a low frequency (8.14% of the colonies) (Fig. 4C).

### IS*CR1* and IS*Vsa3* elements were deleted in XH1815.

**FIG 6:**
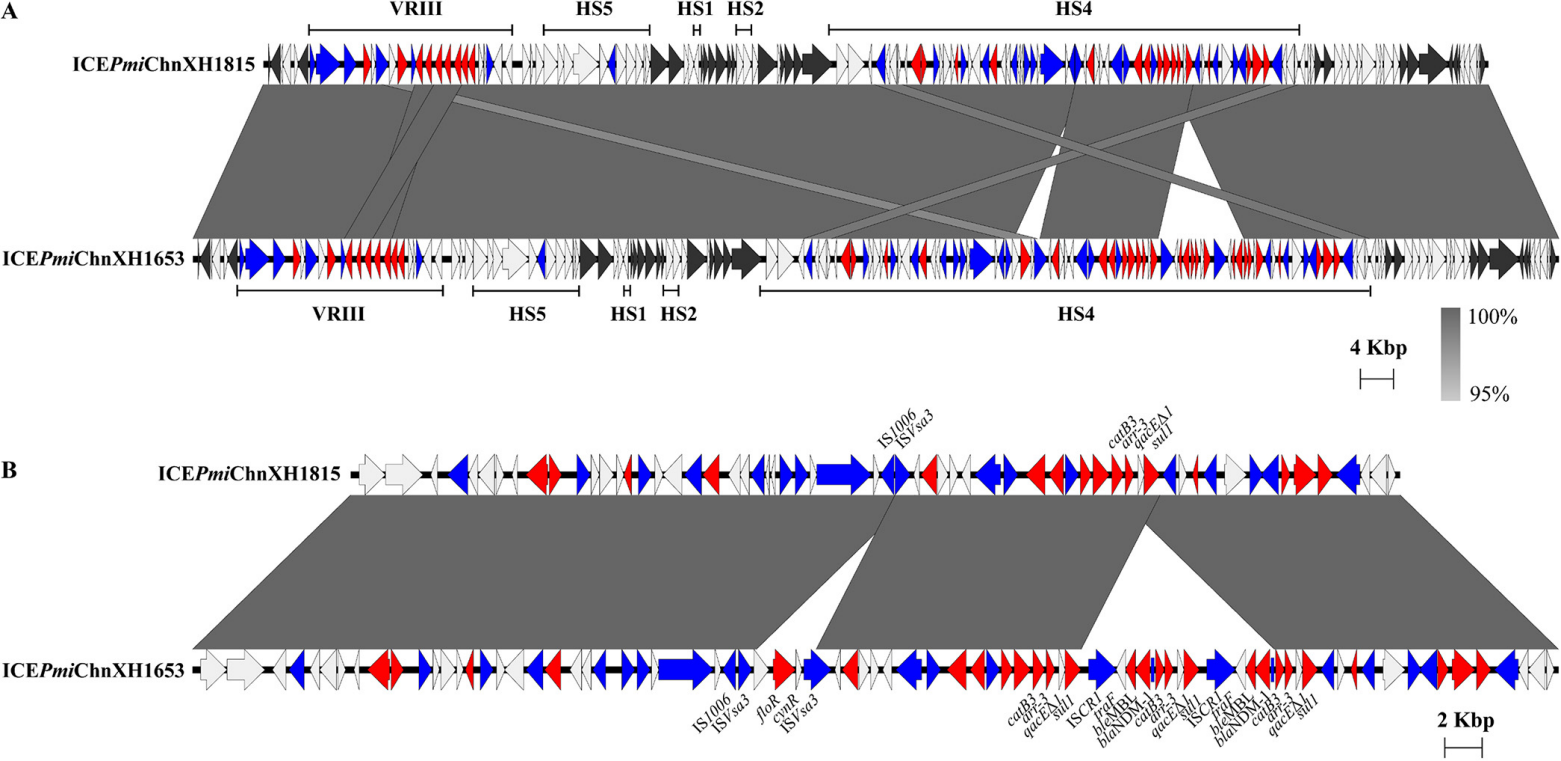
Genetic structure of ICE*Pmi*ChnXH1815. (A) ICE*Pmi*ChnXH1815 compared with ICE*Pmi*ChnXH1653. (B) HS4 of ICE*Pmi*ChnXH1815 compared with HS4 of ICE*Pmi*ChnXH1653. ARGs are in red, transposase or integrase genes are in blue, core genes are in black, and other genes are in white.

The whole genome of XH1815 was sequenced at high accuracy in order to analyze the molecular events that resulted in the deletion of *bla*_NDM-1_. Strain XH1815 was found to harbor the ICE with a size of 148,447 bp, subsequently referred to as ICE*Pmi*ChnXH1815, which chromosomally integrated into the 5′ end of the *prfC* gene. Comparative analysis showed ICE*Pmi*ChnXH1815 to exhibit sequence coverage of 99% and identity of 100% to ICE*Pmi*ChnXH1653. A deletion of two fragments was found in the HS4 region of ICE*Pmi*ChnXH1815 (Fig. 6A) and corresponds to the tandem copies of the IS*CR1* and IS*Vsa3* elements that included the carbapenemase gene *bla*_NDM-1_ and the chloramphenicol ARG *floR*, respectively (Fig. 6B). Our results indicate that ICE*Pmi*ChnXH1653 obtained the carbapenemase gene by IS*CR1*-mediated homologous recombination. As observed in ICE*Pmi*ChnXH1815, only one copy of the IS*Vsa3* was found, and the IS*Vsa3* element (*hp*-*floR-cynR-*IS*Vsa3*) was deleted compared to ICE*Pmi*ChnXH1653. We also detected the circular intermediate of the IS*Vsa3* element, which suggests that IS*Vsa3*-mediated transfer of *folR* had occurred and the circular intermediate *hp*-*floR-cynR-*IS*Vsa3* had inserted at the location of IS*Vsa3*.

## DISCUSSION

The observed increase of P. mirabilis strains that are resistant to carbapenem mediated by the *bla*_NDM-1_ gene is of concern as only a limited number of antimicrobials remain available for clinical therapy (12). In this study, we isolated an XDR P. mirabilis strain, XH1653, from a patient suffering from a urinary tract infection, which was resistant to all tested antibiotics with the exception of aztreonam. qPCR and sequencing analysis showed that XH1653 carried two copies of *bla*_NDM-1_, which are located in a so-far-undescribed SXT/R391 ICE.

Mobile elements are associated with the formation of clusters containing ARGs in which different determinants that lead to the MDR phenotype are found in close genetic proximity (27). Antimicrobial resistance determinants in ICE*Pmi*ChnXH1653 are carried within VRIII and HS4 regions that are integrated within the conserved ICE backbone, and most of them are located within ARG arrays, composed of ARGs or clusters thereof, and mobile genetic elements such as IS elements, transposons, or integrons. The genes *floR*, *strB*, *strA*, and *sul2* are frequently found in the VRIII region of SXT/R391 ICE members (28). Also, *erm(42)* and *aphA* are observed in the VRIII region of ICE*Apl*Chn1 in Actinobacillus pleuropneumoniae (29). Our finding is also the first report of two copies of the resistance gene fragment *strB*-*aphA* in VRIII.

The *bla*_NDM-1_ gene is mainly and widely spread by an IS*Aba125*-bounded composite transposon Tn*125* (9), which is often located on plasmids in *Enterobacteriaceae* (12, 30, 31). Recently, Kong et al. reported that *bla*_NDM-1_ was embedded in a truncated IS*Aba125* composite transposon flanked by IS*26* in the ICE from P. vulgaris (13). However, the genetic environment around *bla*_NDM-1_ in ICE*Pmi*ChnXH1653 is different from the ICE in the study by Kong et al., which revealed two tandem copies of an IS*CR1* element. A 151-bp truncated IS*Aba125* is found upstream of *bla*_NDM-1_, while the IS*CR1* lies downstream of both copies of the *bla*_NDM-1_ gene and is followed by the bleomycin ARG *ble*_MBL_ and the *trpF* gene, which are often identified near *bla*_NDM-1_ (9). The IS*CR1*-like elements might be responsible for the mobilization of *bla*_NDM-1_ via the rolling-circle replication in *Enterobacteriaceae* (30, 32, 33). In our study, we also observed that the two tandem copies of the IS*CR1* element (IS*CR1*-*traF*-*ble*_MBL_-*bla*_NDM-1_-ΔIS*Aba125*-*catB3*-*arr-3*-*qacE*Δ*1*-*sul1*) were lost following extended passage under nonselective conditions, which is a clear indication that two tandem copies of the IS*CR1* element move into the 3′-CS (*qacE*Δ*1*/*sul1*) of a class 1 integron [*aac(6′)-Ib-cr*-*bla*_OXA-1_-*catB3*-*arr3*-*qacE*Δ*1*-*sul1*] by homologous recombination, resulting in the transmission of *bla*_NDM-1_. Homologous recombination is likely to contribute extensively to the duplication of ARGs when no selective pressure is applied. It is possible that the a single IS*CR1* element mobilizes the *bla*_NDM-1_ gene to move into the 3′-CS by rolling-circle transposition and that after attachment, subsequent homologous recombination may result in a duplication of the ARG (34). A similar structure of the two tandem copies of the IS*CR1* element was also observed in the chromosome of E. coli Y5 (GenBank accession no. CP013483) (26), and one copy of the IS*CR1* element appears to be intact in the plasmid from P. mirabilis (GenBank accession no. KP662515), suggesting a potential translocation of the *bla*_NDM-1_ gene between different mobile genetic elements (ICEs and plasmids) and the integration into the chromosome. To the best of our knowledge, this is the first description of two copies of *bla*_NDM-1_ embedded within the IS*CR1* element, not only in the SXT/R391 element but also in P. mirabilis. IS*Vsa3* belongs to the IS*CR2* family; two copies of the IS*Vsa3* in the same orientation were found in the HS4 region of ICE*Pmi*ChnXH1653, which contained the chloramphenicol ARG *floR*. He et al. reported that IS*Vsa3* is able to mediate the transposition of *tet(X)*-carrying cassettes and that the circular intermediate was able to insert at the location of IS*Vsa3* on the plasmid (35). We found that the IS*Vsa3* element (*hp*-*floR-cynR-*IS*Vsa3*) was lost during the serial passage in the laboratory, indicating the IS*Vsa3*-mediated transfer of *floR* had occurred and IS*CR* elements played a major role for the host bacteria in the mobilization and accumulation of antibiotic resistance genes. After the deletion of the IS*CR1* and IS*Vsa3* element in ICE*Pmi*ChnXH1653, the HS4 of the ICE exhibited a high similarity to the HS4 of ICE*Pmi*BCP11 with a coverage of 95% (increase from 82% to 95%) and a sequence identity of 99.95%, contributing to our knowledge of how the dissemination of an IS*Ppu12*-mediated composite MDR transposon in different P. mirabilis strains occurs. A general observation is that the ICE*Pmi*ChnXH1653 has been highly efficient in recruiting antimicrobial resistance traits. It has been suggested that recent MDR members of the SXT/R391 family could have evolved from a common ancestor through stepwise integration of horizontally acquired ARG arrays into the conserved backbone.

Here, we performed an integrative approach to gain insights into different aspects of ICE*Pmi*ChnXH1653 evolution and biology. An intergenus transfer of ICE*Pmi*ChnXH1653 from P. mirabilis to E. coli EC600 at a frequency of 1.5 × 10^−7^ was observed, which is relatively low for SXT/R391 (22). The ICE*Pmi*ChnXH1653 in the recipient strain exhibited a higher excision frequency and extrachromosomal copy number than the ICE in the ancestor strain. As expected for one *attP* site on the circular ICE resulting in one unoccupied *attB* site on the chromosome (*attP/attB* = 1), an increase in the copy number of *attP*/*attB* would indicate that the excised ICE replicates more frequently than the chromosome (36). The extrachromosomal autonomous replication appears to be common for ICEs, as our results for *attP*/*attB* were consistent with previous reports (36, 37). The relatively higher excision frequency in recipient strain EC600 may be caused by a genetic instability after entering a new host cell. We also found that the acquisition of ICE*Pmi*ChnXH1653 results in a fitness cost for the ICE-free recipient strain EC600. Here, several genes showed altered transcription in EC600 after the acquisition of ICE. This number was lower than a report which described that a total of 161 genes were differentially expressed in Pseudomonas putida with the ICE*clc* (38). The presence of the ICE*clc* can influence a number of cellular pathways, resulting in direct benefits but also in indirect costs for P. putida (38). The difference of the impact on the transcription of the bacteria might be caused by the genetic interrelationship of the strains used in the studies: the original host of ICE*clc* is Pseudomonas knackmussii B13, with the new host in the study by Miyazaki et al. (38) belonging to the same genus. However, in our project we used EC600 as the new host for the P. mirabilis XH1653-derived element ICE*Pmi*ChnXH1653. Interestingly, the genes carried in ICE*Pmi*ChnXH1653 showed a higher expression in XH1814 compared to the average expression in EC600 or XH1814, confirming ICE*Pmi*ChnXH1653 activation in XH1814, possibly explaining the burden in fitness.

In conclusion, this is the first report of a novel SXT/R391 ICE carrying two tandem copies of *bla*_NDM-1_. The genetic environment of *bla*_NDM-1_ was identical to that of the previously reported *bla*_NDM-1_-carrying plasmid of P. mirabilis PM58 and chromosome of E. coli Y5. The ICE*Pmi*ChnXH1653 could be transferred between bacterial genera—within the order *Enterobacteriales*—from P. mirabilis to E. coli, indicating that the transmission of *bla*_NDM-1_ by IS*CR1* elements or ICEs may be an important contributor to the carbapenem resistance development across species.

## MATERIALS AND METHODS

### Bacterial strains and susceptibility testing.

P. mirabilis strain XH1653 was isolated in October 2015 from a urine sample of a 49-year-old male patient in a hospital in Zhejiang province, China. All isolates used in this study (Table 2) were cultured in MH agar plates or broth (Oxoid, Hampshire, United Kingdom) and Luria-Bertani (LB) broth (Sangon Biotech, Shanghai, China) at 37°C. The following 21 compounds were tested using the BD Phoenix 100 automated microbiology system (Becton, Dickinson, MD, USA): imipenem, meropenem, gentamicin, amikacin, cefazolin, ceftazidime, cefotaxime, cefepime, ampicillin, piperacillin, amoxicillin-clavulanate, ampicillin-sulbactam, piperacillin-tazobactam, trimethoprim-sulfamethoxazole, ciprofloxacin, chloramphenicol, levofloxacin, moxifloxacin, aztreonam, tetracycline, and colistin. Susceptibility of XH1653, EC600, XH1814, and XH1815 to antibiotics (imipenem, meropenem, gentamicin, cefepime, ciprofloxacin, tetracycline, and trimethoprim-sulfamethoxazole) was also determined by the broth microdilution method. The results of susceptibility testing were interpreted according to the Clinical and Laboratory Standards Institute (CLSI) guidelines (39). E. coli ATCC 25922 served as a control strain.

**TABLE 2:** Bacterial strains used in this study

Strain	Description
XH1653	ICE*Pmi*ChnXH1653-carrying multidrug-resistant P. mirabilis, wild-type strain
XH1814	ICE*Pmi*ChnXH1653-carrying transconjugant, EC600 as the recipient strain
XH1815	Selected isolate of XH1814D10, containing ICE*Pmi*ChnXH1815
EC600	ICE-free recipient strain, rifampin resistance
EC600D5	EC600 population at the 5th day of passage
EC600D10	EC600 population at the 10th day of passage
XH1814D5	XH1814 population at the 5th day of passage
XH1814D10	XH1814 population at the 10th day of passage

### Whole-genome sequencing and sequence analysis.

Genomic DNA was extracted and subjected to whole-genome sequencing using both the Illumina HiSeq and Nanopore MinION platforms at Zhejiang Tianke (Hangzhou, China). Long-read library preparation for Nanopore sequencing was performed with a one-dimensional (1D) sequencing kit (SQK-LSK109; Nanopore). The libraries were sequenced on a MinION device with a 1D flow cell (FlO-MIN106; Nanopore) and base called with Guppy v2.3.5 (Nanopore). Long- and short-read sequence data were used in a hybrid *de novo* assembly using Unicycler v0.4.8 (40), followed by Pilon v1.23 (41). ARGs were identified using the ResFinder database (42) with Abricate 0.8 (https://github.com/tseemann/abricate). The complete nucleotide sequence of ICE in the strain XH1653 was identified by ICEfinder (https://db-mml.sjtu.edu.cn/ICEfinder/) with manual modification (43). Sequence comparisons were performed using BLASTn v2.4.0 (44) and visualized using Easyfig v2.2.3 (45).

### Bacterial conjugations.

Conjugation experiments were carried out by filter mating with the rifampin-resistant E. coli EC600 as recipient. Overnight cultures of XH1653 and EC600 were mixed on an MH plate and incubated at 37°C for 18 h. The cells on the membrane were collected, resuspended in saline solution, and serially diluted before plating. Donors, recipients, and transconjugants were selected on MH agar plates containing 100 mg/liter rifampin and 100 mg/liter ampicillin. The successful transconjugants were identified by MALDI-TOF MS (bioMérieux, France), and the presence of *bla*_NDM-1_, *attL*, *int*, and *attR* as the marker sequences of ICE in transconjugants was determined by PCR (Table 3). The MIC profiles of the transconjugants were determined for differentiation between transconjugants and donor strains. The transconjugant was designated XH1814. The ICE transfer frequency was calculated as the number of transconjugants per donor cell.

**TABLE 3:** Primers used in this study

Target	Primer	Primer sequence (5′–3′)	Amplicon size (bp)
*attL*	C600-LE-1	GTTTCTTCGTTGCACGAACTGG	348
	LE-4	GTACACACTTTCCGAGGTTACG	
*attR*	C600-RE-1	CGGTCTGAATGGCCTGTCCGAA	464
	RE-4	CCGCAATACCCTGCAATACCGA	
*int*	ICE-int-F	CGTAACCTCGGAAAGTGTGTAC	640
	ICE-int-R	TGTGCCACAGCTTGTTTCGTG	
*bla*_NDM-1_	NDM-1-F	TTGCCCAATATTATGCACCC	552
	NDM-1-R	GCCGGGGTAAAATACCTTGA	
*bla*_NDM-1_ (for qPCR)	QNDM-1-F	AACGCATTGGCATAAGTCGC	178
	QNDM-1-R	GATACCGCCTGGACCGATG	
*gyrB* (for qPCR)	QgyrB-F	GCAGCCCACCAGAGACTTTA	192
	QgyrB-R	TCGCGGGTTACTGTGATGAG	
*attB* of EC600 (for qPCR)	QC600-attB-F	CGACTTAGCGTGCTGGTTGG	201
	QC600-attB-R	GCGATGCCGCTTACTCAAGA	
*attB* of XH1653 (for qPCR)	Q1653-attB-F	AGTGCAGTGCATTCACTTGTT	198
	Q1653-attB-R	TTGAGCCACGCCCTTTTACT	
*prfC* of EC600 (for qPCR)	QC600-prfC-F	GTCACCCGCCATAAAGGTCA	194
	QC600-prfC-R	CCGTAGAAGCGAGCGAAGAT	
*prfC* of XH1653 (for qPCR)	Q1653-prfC-F	TGGCCTGCATAATCACGGTA	190
	Q1653-prfC-R	AAACACCTGTACCGCACCTT	
*attP* (for qPCR)	QICE-attP-F	AACACGACGGATTTGACAAGC	221
	QICE-attP-R	ACGTAGAGATGTGATTGTGGTGT	
*int* (for qPCR)	QICE-int-F	TATACGACGCTCTGGCGAAG	192
	QICE-int-R	AAACCATCATCGAGCCGACA	

### Growth rate determination.

Three independent cultures of EC600 and XH1814 were grown overnight and diluted to 1:100 in MH broth, and then aliquots were placed into a flat-bottom 100-well plate in three replicates. The plate was incubated at 37°C with agitation. The optical density at 600 nm (OD_600_) of each culture was continuously determined for 20 h using a Bioscreen C MBR machine (Oy Growth Curves Ab Ltd., Finland). Growth rate was estimated based on OD_600_ curves using an R script as previously described (46), and values returning a *P* value of <0.05 from a Student *t* test were taken as significant.

### Real-time quantitative PCR.

The frequency of excision and mean copy number per cell, extrachromosomal copy number of the ICE, and the copy number of *bla*_NDM-1_ per chromosome were assessed by real-time quantitative PCR (37), using the formula as described previously (47). The genomic DNA was extracted using the QIAamp DNA minikit (Qiagen, USA), and quality and quantity of genomic DNA were determined by a NanoDrop spectrophotometer. Primers are listed in Table 3. Triplicate samples were included in each run, and qPCR experiments were performed in triplicate using TB Green Premix *Ex Taq* II (TaKaRa Bio) in a LightCycler 480 system (Roche, Switzerland).

### RNA-Seq.

Three single colonies of EC600 and XH1814 were cultured overnight at 37°C in MH broth. Strains were diluted 1:100 in 100 ml of fresh MH broth and harvested at the mid-log growth phase. The cells were collected at 4°C using centrifugation (5,000 rpm, 10 min). Total RNA was extracted using TRIzol reagent (Invitrogen, Carlsbad, CA, USA) after liquid nitrogen grinding. Bacterial mRNA sequence library construction and sequencing were performed by Zhejiang Tianke (Hangzhou, China) (48). The sequenced reads were mapped to the EC600 genome and ICE sequence, respectively, using Rockhopper version 2.0.3 (49). The raw read count in output of Rockhopper was analyzed by the edgeR package (50). ggplot2 was used for figure generation (51).

### Experimental evolution under nonselective conditions.

Three single colonies of XH1814 and the EC600 ancestor strain were inoculated in MH broth without antibiotics and cultured under shaking (200 rpm) at 37°C. All evolved lineages were passaged daily. A 20-μl volume of overnight culture was collected and used for inoculation at a 1:100 dilution every day. Growth curves were performed every 5 days to assess the evolutionary changes.

### Detection of carbapenem resistance loss.

XH1814, three lineages of XH1814 at day 5, and three lineages of XH1814 at day 10 were incubated in MH broth at 37°C for 18 h. Overnight cultures were serially diluted before plating and were selected on an MH agar plate without antibiotics or with 0.25 mg/liter meropenem, respectively. The frequency of carbapenem resistance in XH1814 was calculated as the number of cells observed on an MH agar plate containing 0.25 mg/liter meropenem versus cells observed on an MH agar plate without antibiotics. The detection experiments were performed in triplicate.

### Data availability.

The complete genome sequences of P. mirabilis XH1653 and E. coli XH1815 isolates were deposited in GenBank under accession numbers CP065039 and CP069386, respectively. The RNA-Seq data from E. coli EC600 and E. coli XH1814 were deposited in GenBank under BioProject no. PRJNA699923.
