# Supplementary material for: A Novel SXT/R391 Integrative and Conjugative Element Carries Two Copies of the blaNDM-1 Gene in Proteus mirabilis
Source: mSphere. 2021 Aug 11;6(4):e00588-21. doi: 10.1128/mSphere.00588-21 (PMC8386438; doi:10.1128/mSphere.00588-21)
Supplement: TABLE S1 [file msphere.00588-21-st001.pdf]

Table S1. Genes changed significantly in transcriptome.

Synonym	Product	logFC	logCPM	PValue	FDR	KO
DK885_16125	4-oxalomesaconate tautomerase	2.517372	0.566193	4.71E-05	0.008759	
DK885_04865	hypothetical protein	2.056482	0.503417	0.000306	0.024696	
DK885_21420	DUF1190 domain-containing protein	1.844363	0.484804	0.000418	0.029798	
DK885_12690	bifunctional aldehyde dehydrogenase/enoyl-CoA hydratase	1.771809	1.957916	1.11E-05	0.003692	K02618
DK885_12575	hypothetical protein	1.761955	1.363996	0.000731	0.041649	K00981
DK885_06720	hydrogenase-4 component J	1.760796	1.0031	0.000259	0.022637	K12145
DK885_07285	hypothetical protein	1.742248	0.988782	0.000487	0.032024	
DK885_20455	DUF466 domain-containing protein	1.568213	4.218342	0.000134	0.016412	
DK885_04375	galactose-proton symporter	1.242983	8.106981	0.000181	0.018019	K08137
DK885_04690	guanine/hypoxanthine permease GhxQ	-1.01974	5.102868	7.75E-05	0.011835	K06901
DK885_00160	multidrug resistance protein MdtL	-1.0275	3.844364	0.000224	0.020823	K08163
DK885_16205	3-deoxy-7-phosphoheptulonate synthase AroG	-1.03787	7.143511	1.30E-05	0.003692	K01626
DK885_04695	guanine deaminase	-1.03986	5.318978	1.23E-05	0.003692	K01487
DK885_00170	tryptophanase	-1.05668	14.14388	0.000533	0.033498	K01667
DK885_11760	cold-shock protein CspB	-1.19204	2.895234	0.000588	0.034683	K03704
DK885_00175	tryptophanase leader peptide	-1.19962	9.498215	2.51E-05	0.005426	

DK885_06605	exodeoxyribonuclease VII large subunit	-1.23474	7.014753	6.50E-06	0.002781	K03601
DK885_19615	pyruvate dehydrogenase complex repressor	-1.24877	5.260095	1.14E-05	0.003692	K05799
DK885_19665	dephospho-CoA kinase	-1.27567	2.722826	0.000241	0.02149	K00859
DK885_19560	hypothetical protein	-1.3322	2.667981	0.000152	0.016858	
DK885_15005	periplasmic AppA protein	-1.36997	5.428683	0.000162	0.016858	K01093
DK885_11375	nucleoid-associated protein	-1.68226	1.379283	0.000117	0.015127	
